# The genome sequence of the fish leech, *Piscicola geometra* (Linnaeus, 1761)

**DOI:** 10.12688/wellcomeopenres.19488.1

**Published:** 2023-05-26

**Authors:** Jason Doe, Natural History Museum Genome Acquisition Lab, Darwin Tree of Life Barcoding collective, Wellcome Sanger Institute Tree of Life programme, Wellcome Sanger Institute Scientific Operations: DNA Pipelines collective, Tree of Life Core Informatics collective, Darwin Tree of Life Consortium

**Affiliations:** 1 Environment Agency, Tewkesbury, England, UK

**Keywords:** Piscicola geometra, fish leech, genome sequence, chromosomal, Hirudinida

## Abstract

We present a genome assembly from an individual *Piscicola geometra* (the fish leech; Annelida; Clitellata; Hirudinida; Piscicolidae). The genome sequence is 171.1 megabases in span. Most of the assembly is scaffolded into 17 chromosomal pseudomolecules. The mitochondrial genome has also been assembled and is 15.1 kilobases in length.

## Species taxonomy

Eukaryota; Metazoa; Spiralia; Lophotrochozoa; Annelida; Clitellata; Hirudinea; Hirudinida; Oceanobdelliformes; Piscicolidae; *Piscicola*; *Piscicola geometra* (Linnaeus, 1761) (NCBI:txid60958).

## Background

*Piscicola geometra*, the fish leech, is an ectoparasite of freshwater fish. It is widespread in the UK, inhabiting well-oxygenated rivers and still waters that support its fish hosts. In lake Windermere the species completes its lifecycle within 7 to 9 months, allowing for 2 to 3 generations per year ( Elliott & Dobson, 2015).

Piscicolid leeches have large anterior and posterior suckers that are clearly distinct from the rest of the body, which readily separates them from other British leech families. Until 2012, *Piscicola geometra* was assumed to be the sole species of *Piscicola* in Britain. However, *Piscicola* specimens collected in Yorkshire in 2006 were determined to be *Piscicola siddalli*, which was described as a new species in 2012 by Bielecki *et al*. Consequently, it is likely that some British records of *Piscicola geometra* might actually pertain to *P. sidalli*. As of March 2023, the Environment Agency has records from 194 distinct survey sites encompassing most English counties for *Piscicola siddalli*, compared to 4384 distinct survey sites for *P. geometra* (data source: ( Environment Agency, 2021)).

**Figure 1.**
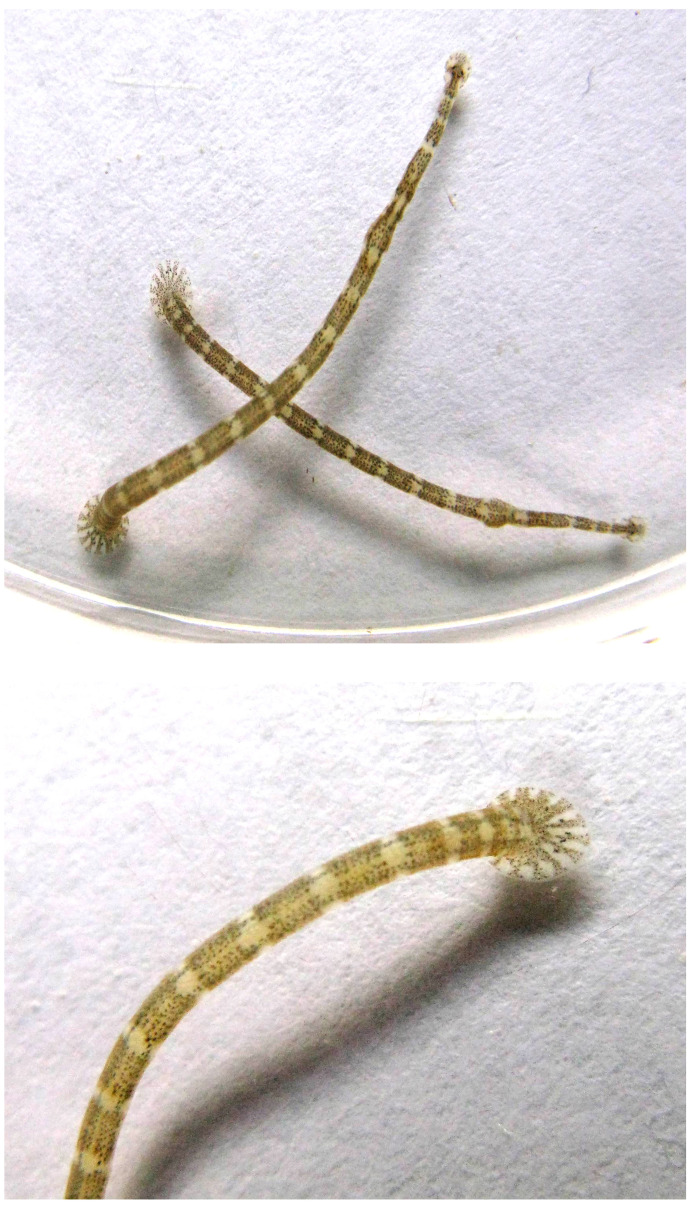
Photograph of a *Piscicola geometra* specimen (not the specimen used for genome sequencing) by Ondřej Zicha (biolib.cz), CC-BY.

Identifying mature, clearly marked specimens of *P. geometra* and *P. siddalii* is straightforward: dorsally *P. geometra* has a large, central diamond shaped spot of pale pigment on most body segments ( Figure 1), compared to 3 to 5 transverse, small, pale spots in *P. siddalli*. Furthermore, there are differences in the number of subdivisions in the body segments, and the body shape, but these features are subtle and can be difficult to interpret ( Bielecki *et al.*, 2012; Elliott & Dobson, 2015).

Here we present a chromosomally complete genome sequence for *Piscicola geometra*, sequenced as part of the Darwin Tree of Life project, based on one specimen collected from Gloucester and Sharpness Canal, England. 

## Genome sequence report

The genome was sequenced from an individual *Piscicola geometra* ( Figure 1) collected from Gloucester and Sharpness Canal, England, UK (latitude 51.86, longitude –2.26). A total of 76-fold coverage in Pacific Biosciences single-molecule HiFi long was generated. Primary assembly contigs were scaffolded with chromosome conformation Hi-C data. Manual assembly curation corrected 43 missing joins or mis-joins and removed 6 haplotypic duplications, reducing the assembly length by 0.61%% and the scaffold number by 15.85%.

**Figure 2.**
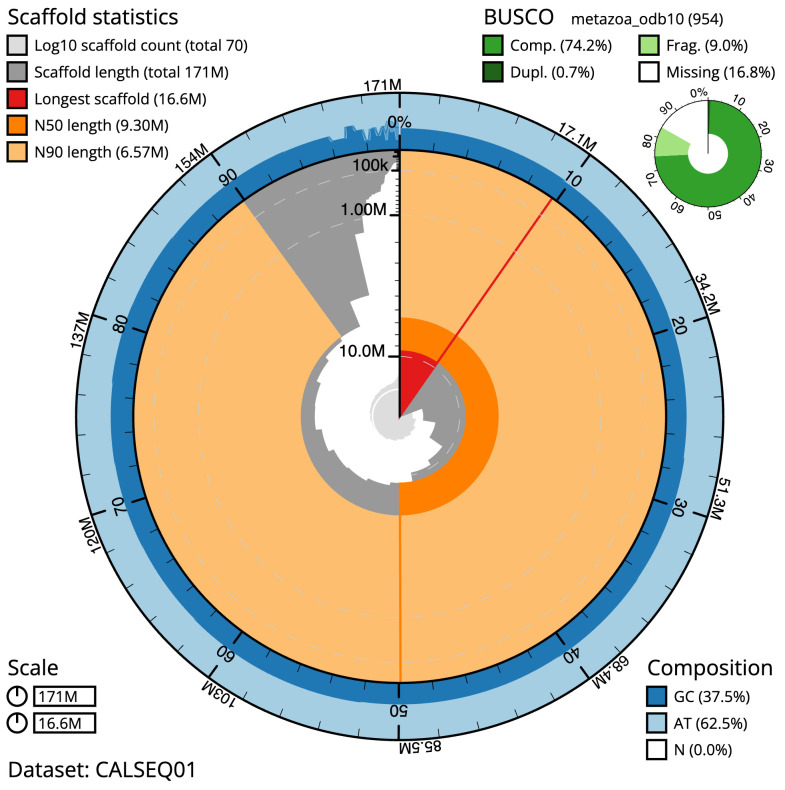
Genome assembly of *Piscicola geometra*, wrPisGeom1.1: metrics. The BlobToolKit Snailplot shows N50 metrics and BUSCO gene completeness. The main plot is divided into 1,000 size-ordered bins around the circumference with each bin representing 0.1% of the 171,095,250 bp assembly. The distribution of scaffold lengths is shown in dark grey with the plot radius scaled to the longest scaffold present in the assembly (16,636,273 bp, shown in red). Orange and pale-orange arcs show the N50 and N90 scaffold lengths (9,301,023 and 6,573,335 bp), respectively. The pale grey spiral shows the cumulative scaffold count on a log scale with white scale lines showing successive orders of magnitude. The blue and pale-blue area around the outside of the plot shows the distribution of GC, AT and N percentages in the same bins as the inner plot. A summary of complete, fragmented, duplicated and missing BUSCO genes in the metazoa_odb10 set is shown in the top right. An interactive version of this figure is available at https://blobtoolkit.genomehubs.org/view/wrPisGeom1.1/dataset/CALSEQ01/snail.

The final assembly has a total length of 171.1 Mb in 69 sequence scaffolds with a scaffold N50 of 9.3 Mb ( Table 1). Most (96.08%) of the assembly sequence was assigned to 17 chromosomal-level scaffolds. Chromosome-scale scaffolds confirmed by the Hi-C data are named in order of size ( Figure 2– Figure 5; Table 2). While not fully phased, the assembly deposited is of one haplotype. Contigs corresponding to the second haplotype have also been deposited.] The mitochondrial genome was also assembled and can be found as a contig within the multifasta file of the genome submission.

**Table 1.** Genome data for *Piscicola geometra*, wrPisGeom1.1.

Project accession data		
Assembly identifier	wrPisGeom1.1	
Species	*Piscicola geometra*	
Specimen	wrPisGeom1	
NCBI taxonomy ID	60958	
BioProject	PRJEB52863	
BioSample ID	SAMEA7521191	
Isolate information	wrPisGeom1, hermaphrodite	
Assembly metrics *		*Benchmark*
Consensus quality (QV)	51.3	*≥ 50*
*k*-mer completeness	99.97%	*≥ 95%*
BUSCO **	C:74.2%[S:73.5%,D:0.7%], F:9.0%,M:16.8%,n:954	*C ≥ 95%*
Percentage of assembly mapped to chromosomes	96.08%	*≥ 95%*
Sex chromosomes	-	*localised homologous pairs*
Organelles	Mitochondrial genome assembled	*complete single alleles*
Raw data accessions		
PacificBiosciences SEQUEL II	ERR9744411	
Hi-C Illumina	ERR9767808	
Genome assembly		
Assembly accession	GCA_943735955.1	
*Accession of alternate haplotype*	GCA_943735945.1	
Span (Mb)	171.1	
Number of contigs	254	
Contig N50 length (Mb)	1.4	
Number of scaffolds	69	
Scaffold N50 length (Mb)	9.3	
Longest scaffold (Mb)	16.6	

* Assembly metric benchmarks are adapted from column VGP-2020 of “Table 1: Proposed standards and metrics for defining genome assembly quality” from ( Rhie *et al.*, 2021). ** BUSCO scores based on the metazoa_odb10 BUSCO set using v5.3.2. C = complete [S = single copy, D = duplicated], F = fragmented, M = missing, n = number of orthologues in comparison. A full set of BUSCO scores is available at https://blobtoolkit.genomehubs.org/view/wrPisGeom1.1/dataset/CALSEQ01/busco.

**Figure 3.**
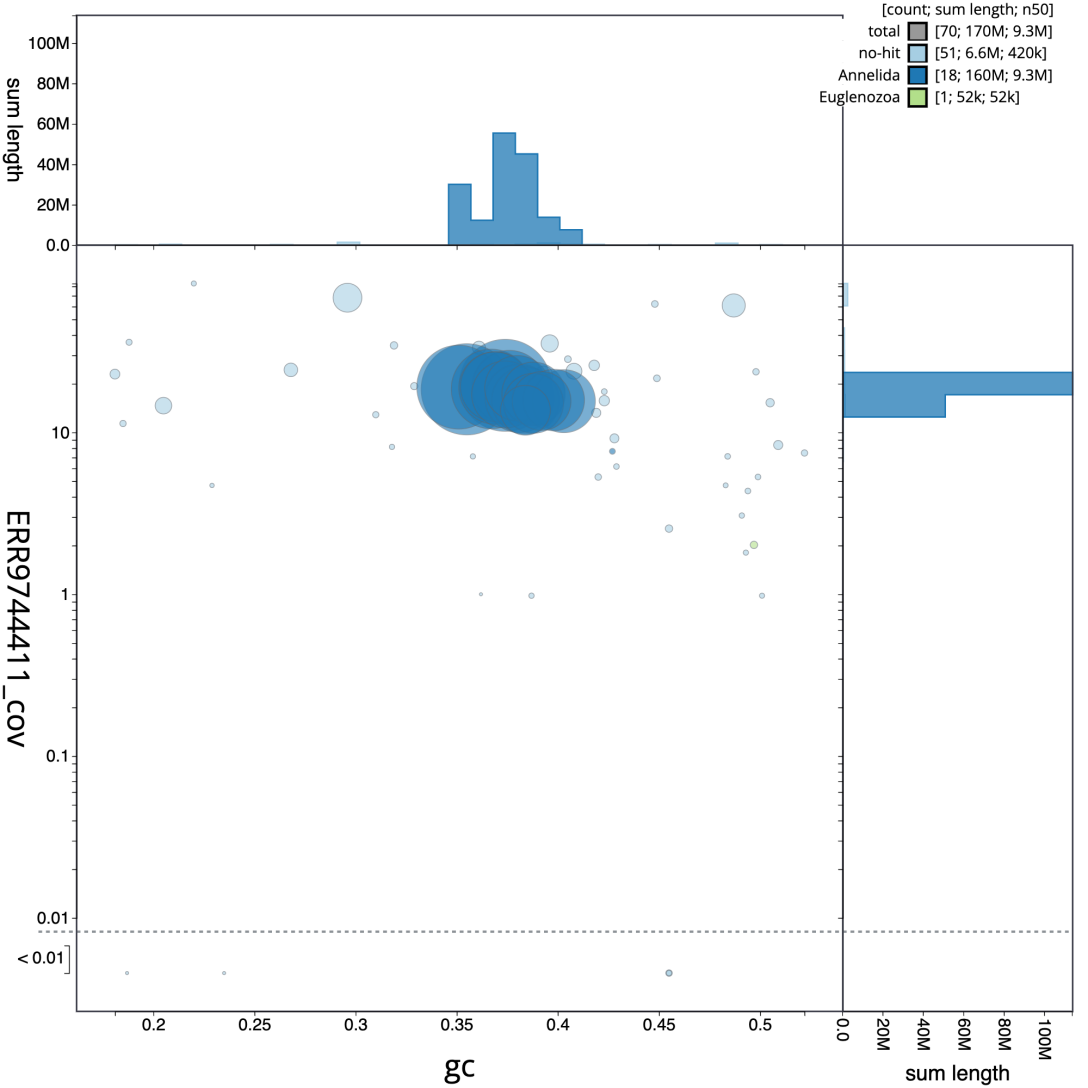
Genome assembly of *Piscicola geometra*, wrPisGeom1.1: BlobToolKit GC-coverage plot. Scaffolds are coloured by phylum. Circles are sized in proportion to scaffold length. Histograms show the distribution of scaffold length sum along each axis. An interactive version of this figure is available at https://blobtoolkit.genomehubs.org/view/wrPisGeom1.1/dataset/CALSEQ01/blob.

**Figure 4.**
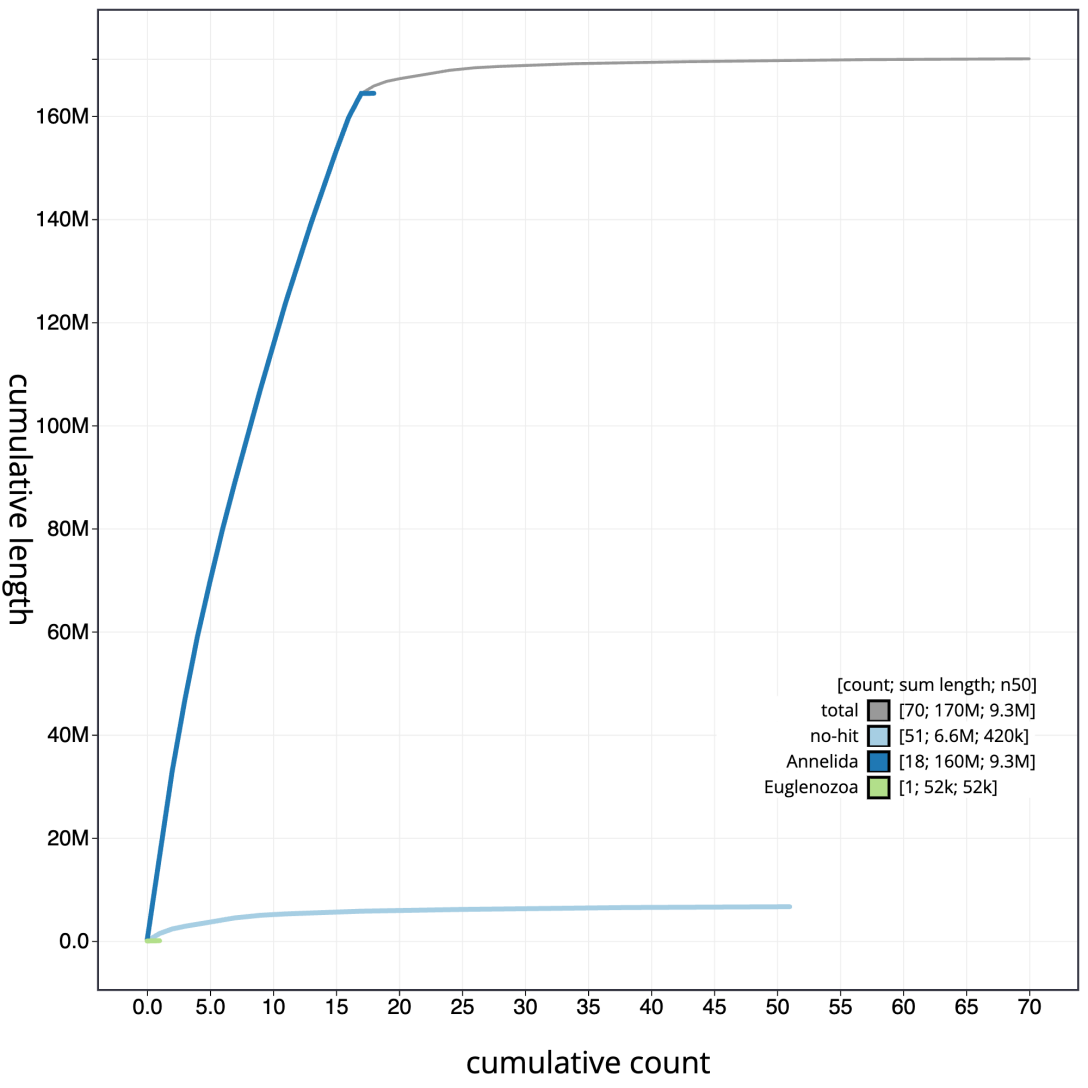
Genome assembly of *Piscicola geometra*, wrPisGeom1.1: BlobToolKit cumulative sequence plot. The grey line shows cumulative length for all scaffolds. Coloured lines show cumulative lengths of scaffolds assigned to each phylum using the buscogenes taxrule. An interactive version of this figure is available at https://blobtoolkit.genomehubs.org/view/wrPisGeom1.1/dataset/CALSEQ01/cumulative.

**Figure 5.**
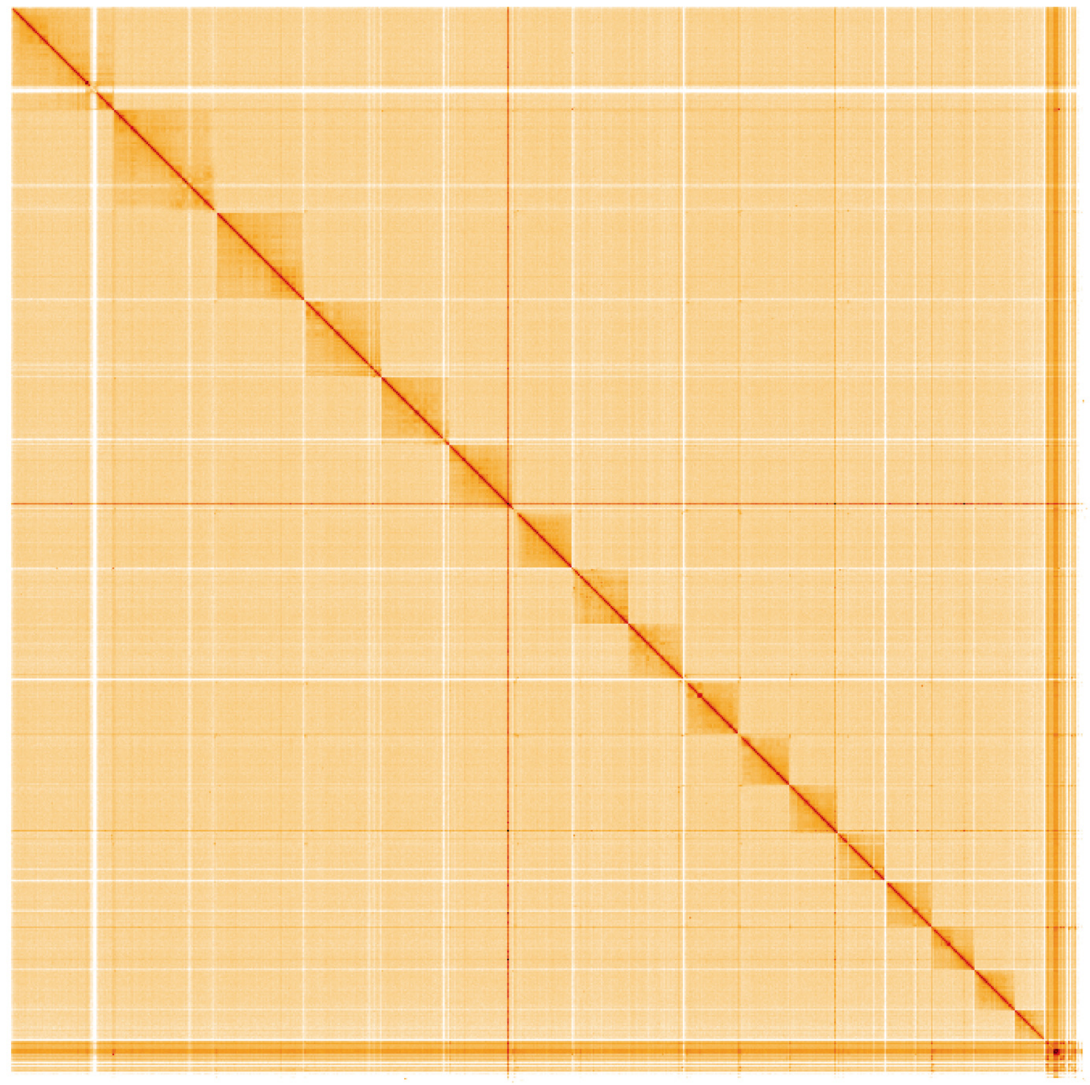
Genome assembly of *Piscicola geometra*, wrPisGeom1.1: Hi-C contact map of the wrPisGeom1.1 assembly, visualised using HiGlass. Chromosomes are shown in order of size from left to right and top to bottom. An interactive version of this figure may be viewed at https://genome-note-higlass.tol.sanger.ac.uk/l/?d=Ye2V71QxQ3qnmuGmSHkfRA.

**Table 2.** Chromosomal pseudomolecules in the genome assembly of *Piscicola geometra*, wrPisGeom1.

INSDC accession	Chromosome	Size (Mb)	GC%
OX030955.1	1	16.64	37.4
OX030956.1	2	16.34	35.5
OX030957.1	3	13.74	35.1
OX030958.1	4	12.34	36.7
OX030959.1	5	10.69	37.6
OX030960.1	6	10.18	36.9
OX030961.1	7	9.3	36.9
OX030962.1	8	9.03	38.1
OX030963.1	9	8.76	38.4
OX030964.1	10	8.64	37.4
OX030965.1	11	8.16	38
OX030966.1	12	7.66	38.8
OX030967.1	13	7.61	40.3
OX030968.1	14	7.22	39.8
OX030969.1	15	6.85	38.8
OX030970.1	16	6.57	39.2
OX030971.1	17	4.65	38.4
OX030972.1	MT	0.02	21.8

The estimated Quality Value (QV) of the final assembly is 51.3 with *k*-mer completeness of 99.97%, and the assembly has a BUSCO v5.3.2 completeness of 74.2% (single = 73.5%, duplicated = 0.7%), using the metazoa_odb10 reference set ( *n* = 954).

Metadata for specimens, spectral estimates, sequencing runs, contaminants and pre-curation assembly statistics can be found at https://links.tol.sanger.ac.uk/species/60958.

## Methods

### Sample acquisition and nucleic acid extraction

A *Piscicola geometra* (specimen no, NHMUK014361516, individual wrPisGeom1) was collected from Gloucester and Sharpness Canal, England, UK (latitude 51.86, longitude –2.26) on 19 March 2019. The specimen was taken from freshwater using a kicknet, and then snap-frozen on dry ice. The specimen was collected and identified by Jason Doe (Environment Agency).

The tissue was prepared and DNA was extracted at the Tree of Life laboratory, Wellcome Sanger Institute (WSI). The wrPisGeom1 sample was weighed and dissected on dry ice with tissue set aside for Hi-C sequencing. Whole organism tissue was cryogenically disrupted to a fine powder using a Covaris cryoPREP Automated Dry Pulveriser, receiving multiple impacts. High molecular weight (HMW) DNA was extracted using the Qiagen MagAttract HMW DNA extraction kit. HMW DNA was sheared into an average fragment size of 12–20 kb in a Megaruptor 3 system with speed setting 30. Sheared DNA was purified by solid-phase reversible immobilisation using AMPure PB beads with a 1.8X ratio of beads to sample to remove the shorter fragments and concentrate the DNA sample. The concentration of the sheared and purified DNA was assessed using a Nanodrop spectrophotometer and Qubit Fluorometer and Qubit dsDNA High Sensitivity Assay kit. Fragment size distribution was evaluated by running the sample on the FemtoPulse system.

### Sequencing

Pacific Biosciences HiFi circular consensus DNA sequencing libraries were constructed according to the manufacturers’ instructions. DNA sequencing was performed by the Scientific Operations core at the WSI on Pacific Biosciences SEQUEL II (HiFi) instrument. Hi-C data were also generated from tissue of wrPisGeom1 that had been set aside, using the Arima2 kit and sequenced on the HiSeq X Ten instrument.

### Genome assembly, curation and evaluation

Assembly was carried out with Hifiasm ( Cheng *et al.*, 2021) and haplotypic duplication was identified and removed with purge_dups ( Guan *et al.*, 2020). The assembly was scaffolded with Hi-C data ( Rao *et al.*, 2014) using YaHS ( Zhou *et al.*, 2023). The assembly was checked for contamination as described previously ( Howe *et al.*, 2021). Manual curation was performed using HiGlass ( Kerpedjiev *et al.*, 2018) and Pretext ( Harry, 2022). The mitochondrial genome was assembled using MitoHiFi ( Uliano-Silva *et al.*, 2022), which runs MitoFinder ( Allio *et al.*, 2020) or MITOS ( Bernt *et al.*, 2013) and uses these annotations to select the final mitochondrial contig and to ensure the general quality of the sequence.

A Hi-C map for the final assembly was produced using bwa-mem2 ( Vasimuddin *et al.*, 2019) in the Cooler file format ( Abdennur & Mirny, 2020). To assess the assembly metrics, the *k*-mer completeness and QV consensus quality values were calculated in Merqury ( Rhie *et al.*, 2020). This work was done using Nextflow ( Di Tommaso *et al.*, 2017) DSL2 pipelines “sanger-tol/readmapping” ( Surana *et al.*, 2023a) and “sanger-tol/genomenote” ( Surana *et al.*, 2023b). The genome was analysed within the BlobToolKit environment ( Challis *et al.*, 2020) and BUSCO scores ( Manni *et al.*, 2021; Simão *et al.*, 2015) were calculated.

Table 3 contains a list of relevant software tool versions and sources.

**Table 3.** Software tools: versions and sources.

Software tool	Version	Source
BlobToolKit	3.4.0	https://github.com/blobtoolkit/blobtoolkit
BUSCO	5.3.2	https://gitlab.com/ezlab/busco
Hifiasm	0.16.1-r375	https://github.com/chhylp123/hifiasm
HiGlass	1.11.6	https://github.com/higlass/higlass
Merqury	MerquryFK	https://github.com/thegenemyers/MERQURY.FK
MitoHiFi	2	https://github.com/marcelauliano/MitoHiFi
PretextView	0.2	https://github.com/wtsi-hpag/PretextView
purge_dups	1.2.3	https://github.com/dfguan/purge_dups
sanger-tol/genomenote	v1.0	https://github.com/sanger-tol/genomenote
sanger-tol/readmapping	1.1.0	https://github.com/sanger-tol/readmapping/tree/1.1.0
YaHS	yahs-1.1.91eebc2	https://github.com/c-zhou/yahs

### Legal and ethical review process for Darwin Tree of Life Partner submitted materials

The materials that have contributed to this genome note have been supplied by a Darwin Tree of Life Partner.

The submission of materials by a Darwin Tree of Life Partner is subject to the **‘Darwin Tree of Life Project Sampling Code of Practice’**, which can be found in full on the Darwin Tree of Life website here. By agreeing with and signing up to the Sampling Code of Practice, the Darwin Tree of Life Partner agrees they will meet the legal and ethical requirements and standards set out within this document in respect of all samples acquired for, and supplied to, the Darwin Tree of Life Project.

Further, the Wellcome Sanger Institute employs a process whereby due diligence is carried out proportionate to the nature of the materials themselves, and the circumstances under which they have been/are to be collected and provided for use. The purpose of this is to address and mitigate any potential legal and/or ethical implications of receipt and use of the materials as part of the research project, and to ensure that in doing so we align with best practice wherever possible.

The overarching areas of consideration are:

- Ethical review of provenance and sourcing of the material
- Legality of collection, transfer and use (national and international)

Each transfer of samples is further undertaken according to a Research Collaboration Agreement or Material Transfer Agreement entered into by the Darwin Tree of Life Partner, Genome Research Limited (operating as the Wellcome Sanger Institute), and in some circumstances other Darwin Tree of Life collaborators.

## Data Availability

European Nucleotide Archive: *Piscicola geometra*. Accession number PRJEB52863; https://identifiers.org/ena.embl/PRJEB52863. ( Wellcome Sanger Institute, 2022) The genome sequence is released openly for reuse. The *Piscicola geometra* genome sequencing initiative is part of the Darwin Tree of Life (DToL) project. All raw sequence data and the assembly have been deposited in INSDC databases. The genome will be annotated using available RNA-Seq data and presented through the Ensembl pipeline at the European Bioinformatics Institute. Raw data and assembly accession identifiers are reported in Table 1.

## Author information

Members of the Natural History Museum Genome Acquisition Lab are listed here:

https://doi.org/10.5281/zenodo.4790042.

Members of the Darwin Tree of Life Barcoding collective are listed here: https://doi.org/10.5281/zenodo.4893703.

Members of the Wellcome Sanger Institute Tree of Life programme are listed here: https://doi.org/10.5281/zenodo.4783585.

Members of Wellcome Sanger Institute Scientific Operations: DNA Pipelines collective are listed here: https://doi.org/10.5281/zenodo.4790455.

Members of the Tree of Life Core Informatics collective are listed here: https://doi.org/10.5281/zenodo.5013541.

Members of the Darwin Tree of Life Consortium are listed here: https://doi.org/10.5281/zenodo.4783558.
