# Supplementary material for: The genome sequence of the fish leech, Piscicola geometra (Linnaeus, 1761)
Source: Wellcome Open Res. 2023 May 26;8:229. [Version 1] doi: 10.12688/wellcomeopenres.19488.1 (PMC11134150; doi:10.12688/wellcomeopenres.19488.1)
Supplement: Peer review history

# Reviewer report: Reviewer response for version 1

**DOI:** 10.21956/wellcomeopenres.21586.r84073

**Published:** 2024-05-28

**Authors:** José María Martín-Durán

**Affiliations:** 1 School of Biological and Behavioural Sciences, Queen Mary University of London, London, England, UK

This genome note reports the sequencing and assembly of the genome of the fish leech *Piscicola geometra*, a broadly distributed fish parasite. The genome meets the high-standards of the Darwin Tree of Life and will help decisively in understanding the biology, diversity and population structure of this parasite of ecological and economic importance.

 Strengths:

 - High-quality chromosome-level genome assembly generated with gold-standard approaches.

 - As an important fish parasite, this is a species that has an ecological and economic impact.

 Weaknesses:

 - The genome has no annotation. It might be helpful to highlight this will come soon.

Are sufficient details of methods and materials provided to allow replication by others?

Yes

Is the rationale for creating the dataset(s) clearly described?

Yes

Are the datasets clearly presented in a useable and accessible format?

Yes

Are the protocols appropriate and is the work technically sound?

Yes

Reviewer Expertise:

Comparative genomics, invertebrate evolutionary and developmental biology, annelid genomics

I confirm that I have read this submission and believe that I have an appropriate level of expertise to confirm that it is of an acceptable scientific standard.

# Reviewer report: Reviewer response for version 1

**DOI:** 10.21956/wellcomeopenres.21586.r59021

**Published:** 2023-08-04

**Authors:** Qingyou Liu

**Affiliations:** 1 Guangdong Provincial Key Laboratory of Animal Molecular Design and Precise Breeding, School of Life Science and Engineering, Foshan University, Foshan, Guangdong, China

DNA sequencing was performed by the Scientific Operations core at the WSI on Pacific Biosciences SEQUEL II (HiFi) instrument. Hi-C data were also generated from tissue of wrPisGeom1 that had been set aside, using the Arima2 kit and sequenced on the HiSeq X Ten instrument. The methods used in this article are suitable.

 The manuscript is well written, interesting and meaningful. The fish leech genome is useful. The genome sequence is 171.1 megabases in span. Most of the assembly is scaffolded into 17 chromosomal pseudomolecules. The mitochondrial genome has also been assembled and is 15.1 kilobases in length. It can be Approved.

Are sufficient details of methods and materials provided to allow replication by others?

Yes

Is the rationale for creating the dataset(s) clearly described?

Yes

Are the datasets clearly presented in a useable and accessible format?

Yes

Are the protocols appropriate and is the work technically sound?

Yes

Reviewer Expertise:

Animal genome and functional genome

I confirm that I have read this submission and believe that I have an appropriate level of expertise to confirm that it is of an acceptable scientific standard.
